# A case of gastrointestinal perforation following transarterial embolization for an intramural hematoma after cold snare polypectomy of an adenoma in the transverse colon

**DOI:** 10.1002/deo2.70017

**Published:** 2024-09-29

**Authors:** Yuu Kodama, Yuji Mizokami, Yuzo Toyama, Hiroyasu Kusaka, Gen Maeda, Shingo Asahara, Ryuji Nagahama, Shin‐ichiro Horiguchi, Hiroki Aoyama

**Affiliations:** ^1^ Department of Gastroenterology New Tokyo Hospital Chiba Japan

**Keywords:** cold snare polypectomy, hematoma, intestinal perforation, post‐operative complications, trans catheter embolization

## Abstract

We encountered a case of a large hematoma developing with perforation shortly after a cold snare polypectomy for a colorectal adenoma. The patient underwent cold snare polypectomy for a 3‐mm type Is lesion in the transverse colon at another facility. Two hours later, she visited the emergency room due to abdominal pain. Contrast‐enhanced computed tomography revealed a 70 mm, high‐intensity mass in the transverse colon with contrast extravasation. We attempted transcatheter arterial embolization to stop the bleeding. Several hours later, the anemia had not worsened, but the severe abdominal pain persisted. Urgent laparoscopic right hemicolectomy was performed due to the possibility of gastrointestinal perforation. The surgery was successfully completed. Pathology reports confirmed the presence of an intramural hematoma in the proximal transverse colon with hemorrhagic infiltration of all layers, along with extensive ischemic changes. A perforation was identified in this area, with mucosal defects observed near the hole, possibly due to cold snare polypectomy.

## INTRODUCTION

Cold snare polypectomy (CSP) has become widely used as a safe technique for removing colorectal lesions <10 mm in size. In this case, following a CSP, a significant hematoma developed, and we subsequently performed trans catheter embolization to manage the bleeding. However, it was later discovered that a perforation had occurred.

## CASE REPORT

**FIGURE 1:**
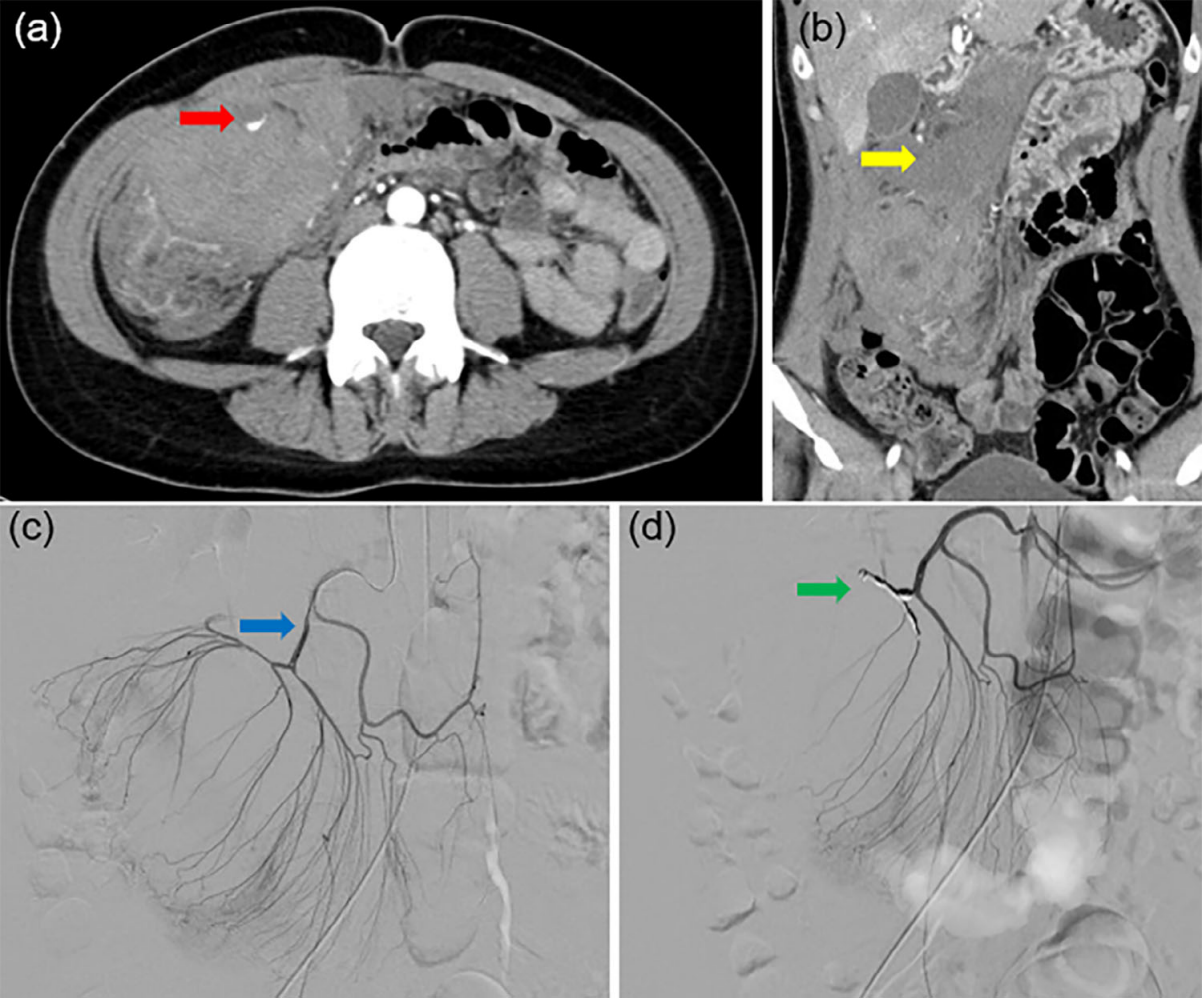
Contrast‐enhanced computed tomography and transcatheter embolization: (a) a 70 mm, hematoma in the transverse colon with contrast extravasation (red arrow), the outer layer of the bowel in the hematoma‐forming region was not discernible; (b) coronal view of high attenuation ascites (yellow arrow); (c) middle colic arteriography couldn't show the bleeding point, but it was considered the vascular supply route (blue arrow); (d) coil embolization was performed on a branch of the middle colic artery for the purpose of preventing rebleeding (green arrow).

**FIGURE 2:**
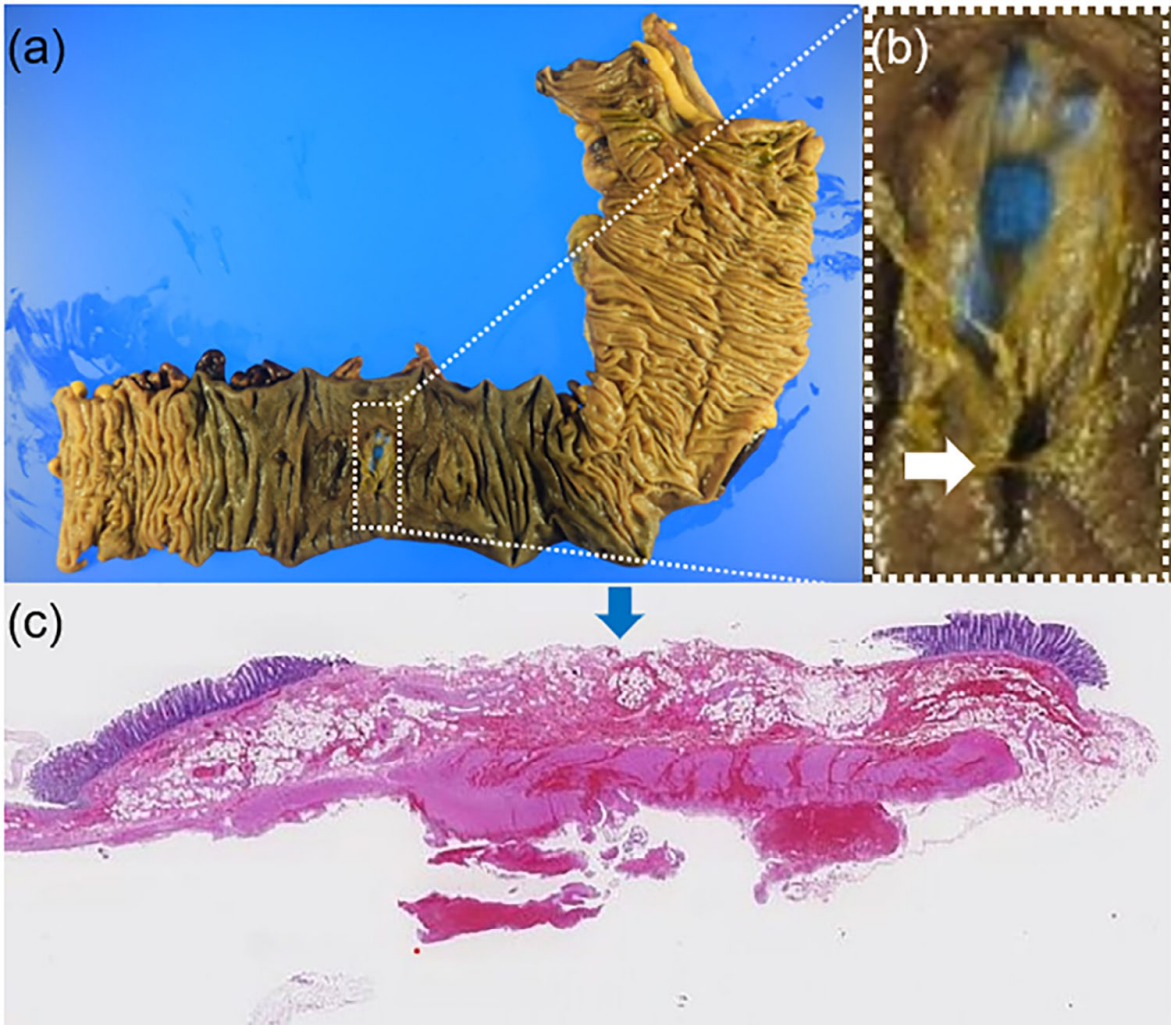
(a) Resected specimen; (b) Depressed lesion near the perforation (white arrow); (c) Hematoxylin and eosin staining of depressed lesion, mucosal defects possibly due to cold snare polypectomy (blue arrow)

**FIGURE 3:**
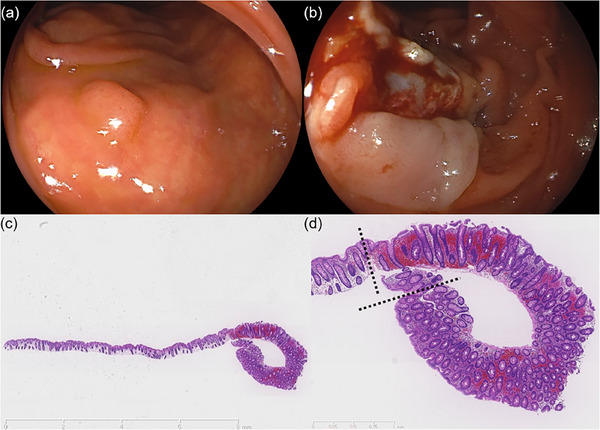
Endoscopic imaging findings and hematoxylin‐eosin staining: (a) 3 mm type 0‐Is lesion detected at the transverse colon; (b) extensive mucosa resection, but no active bleeding immediately after cold snare polypectomy; (c) The cold snare polypectomy specimens seized 12 mm and did not contain muscuralis mucosa and submucosa (5x); (d) The adenoma, which is 4 mm in size, is marked by the black dotted line that represents the boundary between the adenoma and non‐adenoma tissue. Localized microhemorrhages were also observed in the mucosa. (10x)

A 43‐year‐old woman underwent a colonoscopy (ECL‐600ZP7; FUJIFILM) for screening at another facility without anesthesia. Her medical history was unremarkable, with no history of hemodialysis, normal platelet count, no medication use, no coagulation abnormalities, and no colonic diverticula. During the procedure, a 3 mm type 0‐Is lesion in the transverse colon was treated using CSP (CS3‐11023230; Micro‐Tec). The endoscopist who performed the procedure has 4 years of experience. The total time required for the endoscopic examination, including the procedure, was 20 min. There were no adverse events observed during the procedure, such as bleeding or perforation. After the procedure, the patient experienced no symptoms and was discharged immediately. However, two hours later, the patient reported pain that rapidly worsened and subsequently visited our emergency room. The patient complained of abdominal pain alone, without any observation of bloody stools. Upon initial assessment, her vital signs were stable with a temperature of 37.0°C, pulse rate of 74/min, blood pressure of 118/72 mmHg, and respiratory rate of 16/min. Her body mass index was 20.7. Physical examination revealed tenderness in the right upper quadrant upon palpation with muscular defense. Laboratory studies were within normal limits except for an elevated white blood cell count of 14,000/µL, C‐reactive protein level of 0.075 mg/dL, and D‐dimer level of 0.88 µg/mL. Contrast‐enhanced computed tomography (CE‐CT) revealed a 70 mm, high‐intensity mass in the transverse colon with contrast extravasation, suggestive of a bowel wall hematoma (Figure 1a). Additionally, high‐attenuation ascites were observed in the abdominal cavity. The attenuation values of the ascites measured between 25.0 and 59.0 Hounsfield Units (HU). Based on these HU values, the ascites were determined to be either hemorrhagic or indicative of a blood clot. There was slight inflammation around the transverse colon, and the outer layer of the bowel in the hematoma‐forming region was not discernible. Given the indistinct outer layer of the hematoma and the presence of high attenuation ascites suggestive of hemorrhagic fluid, the possibility of the hematoma penetrating the intestinal wall was considered. However, no free air was observed, leading to the determination that there was no gastrointestinal perforation at this stage. Two hours later, the serum hemoglobin level decreased by 1.3 mg/dL, indicating active bleeding. Therefore, transcatheter arterial embolization (TAE) was attempted to stop the bleeding. Although the bleeding point could not be identified on arteriography, CE‐CT suggested a peripheral branch of the middle colic artery, and TAE was performed on it. Several hours later, the anemia had not worsened, but severe abdominal pain with muscular defense persisted. A joint conference between the gastroenterology and gastrointestinal surgery departments was held. Due to the persistence of severe abdominal pain with muscular defense and the presence of high attenuation ascites suggestive of hemorrhagic fluid, the possibility of the hematoma penetrating the intestinal wall at some location was considered. Although the exact site of penetration was unclear, it was presumed to be at a location where the outer layer of the hematoma was indistinct. Consequently, an urgent laparoscopic right hemicolectomy was performed. A huge hematoma was noted in the right transverse colon, and bloody ascites were observed within the abdominal cavity. However, no indications of contamination were detected, and no perforations were confirmed during the surgery. The surgery was successfully completed, and the patient was discharged 14 days later with satisfactory progress. Pathological examination confirmed the presence of an intramural hematoma in the proximal transverse colon with hemorrhagic infiltration of all layers, along with extensive necrotic changes. A perforation was identified in this area, and mucosal defects near the hole were observed, possibly attributed to CSP (Figure 2a–c). Endoscopic imaging and pathological specimens of CSP were obtained from the facility where the procedure was performed and further investigated. Endoscopic imaging of the CSP revealed extensive mucosal resection but no evidence of active bleeding (Figure 3a,b). The CSP specimens measured 12 mm, of which the adenoma was 4 mm, and it did not contain muscularis mucosa and submucosa (Figure 3c,d). Localized microhemorrhages were also observed in the mucosa (Figure 3d).

## DISCUSSION

Intramural hematoma of the colon is a rare occurrence, most frequently associated with blunt trauma. Other risk factors include anticoagulant therapy, bleeding diathesis, malignancies, chemotherapy, vasculitis, collagen diseases, and colonic diverticular disease. However, in this case, no such risk factors were identified. Considering the presence of a mucosal defect potentially caused by CSP within the hematoma, we classify this hematoma as a complication stemming from CSP. Despite the generally low incidence, there have been some reports of hematoma and perforation following CSP. A literature search on PubMed from inception to July 2024 yielded two case reports of hematoma,^1^, ^2^ one associated with anticoagulant therapy and the other without identifiable risk factors. Both cases presented with bloody stools and were managed conservatively. I found one more case through a search on CiNii.^3^ In a 2024 paper, the hematoma ruptured into the abdominal cavity, causing intra‐abdominal hemorrhage. No perforation was observed. This case required surgical intervention. Regarding perforation, three case reports were identified,^4^, ^5^, ^6^ with two cases involving the use of a snare with cautery and two utilizing a specialized cold snare. Perforations were identified during the procedure in three cases, while one case experienced delayed perforation. Notably, none of these cases presented with concurrent hematoma (Table 1). To our knowledge, there are no reports of complications resulting from a combination of hematoma and perforation after CSP.

**TABLE 1:** Reports of complications after cold snare polypectomy.

Ref	Complication	Year	Age (years)	Sex	Location	Polyp size	Device	Time to complication	Treatment
[1]	Hematoma	2023	81	F	Ascending colon	7 mm	N/A	Following day	Conservative treatment
[2]	Hematoma	2022	61	F	Descending colon	8 mm	Cold snare	Following day	Endoscopic detachable snare ligation
[3]	Intra‐abdominal hemorrhage	2024	51	M	Transverse colon	5 mm	Cold snare	Two hours	Surgery
[4]	Perforation	2023	77	M	Transverse colon	3 mm	Cold snare	Six days	Surgery
[5]	Perforation	2019	N/A	N/A	N/A	10 mm 15 mm	Cautery snare	Post‐procedural	Endoscopic clipping
[6]	Perforation	2022	62	F	Sigmoid colon	6 mm	Cold snare	Post‐procedural	Endoscopic clipping

Arakane et al. and Ikegami et al. proposed that pulling the device for resection while snaring the lesion may contribute to hematoma formation.^1^, ^3^ Arimoto et al. refer to this technique as “force cold snare polypectomy”.^7^ Force cold snare polypectomy is often utilized when the snared specimen cannot be resected. It was suggested that by pulling the snare into the scope, the bowel is excessively stretched inward, which may cause a rupture of the deep vessels and result in the formation of a large hematoma.^1^, ^3^ According to the treating physician, force cold snare polypectomy was utilized in this case, potentially contributing to hematoma formation. Therefore, when specimens cannot be removed with CSP, the device should not be pulled indiscriminately.

While the exact cause of the perforation has not been identified, histopathological examination revealed significant ischemic changes and necrotic changes across all layers, suggesting that these changes may be the cause of the perforation. It is possible that intestinal ischemia and necrosis were induced by factors such as the enlargement of a hematoma or transarterial embolization. Three cases of gastrointestinal perforation secondary to huge intramural hematoma of the colon have been identified.^8^, ^9^, ^10^ In all cases, the histopathological findings revealed extensive intramural hemorrhage and widespread necrosis. Sakamoto et al. mention the possibility that ischemia was induced and led to perforation due to the enlargement of a hematoma.^8^ It is known that bowel ischemia induced by TAE can occasionally lead to intestinal perforation. In this case, the bowel ischemia and intestinal perforation may have been caused by an enlargement of the hematoma, TAE, or a combination of these factors.

The timing of the surgery for this case was deemed appropriate. Although no contamination of the ascitic fluid was observed, the perforation meant that fecal matter could have leaked at any time. The absence of free air despite the perforation was likely due to the hematoma covering it. While conservative treatment might have been effective, if the hematoma had been absorbed without the perforation site being sealed, the fecal matter could have leaked from the perforation site, necessitating a larger surgery involving the creation of a stoma.

In conclusion, we experienced a case of gastrointestinal perforation following transarterial embolization for an intramural hematoma after a CSP of an adenoma in the transverse colon. CSP is generally a procedure with few complications; however, it can potentially result in severe complications.

## CONFLICT OF INTEREST STATEMENT

None.

## ETHICS STATEMENT

Approval of the Research Protocol by an Institutional Review Board: N/A.

Informed Consent: Informed consent was obtained from the patients for publication of this case report and any accompanying images.

Registry and the Registration No. of the Study/Trial: N/A.

Animal Studies: N/A.
